# Ethnicity and excess mortality in severe mental illness: a cohort study

**DOI:** 10.1016/S2215-0366(17)30097-4

**Published:** 2017-05

**Authors:** Jayati Das-Munshi, Chin-Kuo Chang, Rina Dutta, Craig Morgan, James Nazroo, Robert Stewart, Martin J Prince

**Affiliations:** a Department of Health Services and Population Research, Institute of Psychiatry, Psychology & Neuroscience, King's College London, London, UK; b Academic Department of Psychological Medicine, King's College London, London, UK; c Cathie Marsh Institute for Social Research, University of Manchester, Manchester, UK

## Abstract

**Background:**

Excess mortality in severe mental illness (defined here as schizophrenia, schizoaffective disorders, and bipolar affective disorders) is well described, but little is known about this inequality in ethnic minorities. We aimed to estimate excess mortality for people with severe mental illness for five ethnic groups (white British, black Caribbean, black African, south Asian, and Irish) and to assess the association of ethnicity with mortality risk.

**Methods:**

We conducted a longitudinal cohort study of individuals with a valid diagnosis of severe mental illness between Jan 1, 2007, and Dec 31, 2014, from the case registry of the South London and Maudsley Trust (London, UK). We linked mortality data from the UK Office for National Statistics for the general population in England and Wales to our cohort, and determined all-cause and cause-specific mortality by ethnicity, standardised by age and sex to this population in 2011. We used Cox proportional hazards regression to estimate hazard ratios and a modified Cox regression, taking into account competing risks to derive sub-hazard ratios, for the association of ethnicity with all-cause and cause-specific mortality.

**Findings:**

We identified 18 201 individuals with a valid diagnosis of severe mental illness (median follow-up 6·36 years, IQR 3·26–9·92), of whom 1767 died. Compared with the general population, age-and-sex-standardised mortality ratios (SMRs) in people with severe mental illness were increased for a range of causes, including suicides (7·65, 95% CI 6·43–9·04), non-suicide unnatural causes (4·01, 3·34–4·78), respiratory disease (3·38, 3·04–3·74), cardiovascular disease (2·65, 2·45–2·86), and cancers (1·45, 1·32–1·60). SMRs were broadly similar in different ethnic groups with severe mental illness, although the south Asian group had a reduced SMR for cancer mortality (0·49, 0·21–0·96). Within the cohort with severe mental illness, hazard ratios for all-cause mortality and sub-hazard ratios for natural-cause and unnatural-cause mortality were lower in most ethnic minority groups relative to the white British group.

**Interpretation:**

People with severe mental illness have excess mortality relative to the general population irrespective of ethnicity. Among those with severe mental illness, some ethnic minorities have lower mortality than the white British group, for which the reasons deserve further investigation.

**Funding:**

UK Health Foundation and UK Academy of Medical Sciences.

## Introduction

The association between severe mental illness (defined here as schizophrenia, schizoaffective disorders, and bipolar affective disorders) and excess mortality has been well established worldwide. Such mortality is not restricted to suicide mortality but also encompasses mortality from natural causes, including cardiovascular and respiratory diseases.^1^

Few studies have assessed the nature of this inequality by ethnicity. In most studies, ethnicity is treated as a confounder or the sample size has been too small to allow stratified analysis. This concern is noteworthy because many mortality risk factors implicated in severe mental illness, such as cardiovascular disease and diabetes,^2^, ^3^ are also known to be more prevalent in some ethnic minority groups relative to white British, European, and non-Hispanic white American populations. Contrary to expectation, results from a 2015 study from the USA^4^ implicated lower all-cause, natural-cause, and unnatural-cause mortality in ethnic minority groups (black non-Hispanic, Hispanic, and other ethnic groups) with schizophrenia than in the white non-Hispanic group with schizophrenia. These findings are yet to be replicated outside the USA.

In this study, we aimed to estimate the risk of all-cause, natural-cause, and unnatural-cause mortality for black Caribbean, black African, south Asian, Irish, and white British people with severe mental illness relative to the general population of England and Wales, and to assess the association of ethnicity and other clinical and sociodemographic factors with mortality risk. The present analysis forms part of a larger investigation into ethnic minority inequalities in severe mental illness.^5^

## Methods

### Study setting and participants

In this longitudinal cohort study, we included individuals with a valid diagnosis of severe mental illness from the case registry of the South London and Maudsley Trust (SLaM), a large secondary care mental health trust serving roughly 1·36 million people in an ethnically diverse catchment area of London, UK.^6^, ^7^ Since 2006, electronic health records have been used for routine patient care in the Trust. The SLaM Clinical Record Interactive Search system, established in 2008, allows search and retrieval of fully anonymised patient records for secondary analysis.^6^, ^7^ Electronic health records predating its establishment have also been incorporated into the system.^7^

**Research in context**

**Evidence before this study**

To assess the evidence for excess mortality in severe mental illness (defined here as schizophrenia, schizoaffective disorders, and bipolar affective disorders) in ethnic minority groups, we systematically searched MEDLINE, PsycINFO, and Embase from database inception to Aug 23, 2016, using the search term “((psychosis OR schizo* OR bipolar) AND (ethnic* OR race OR racial) AND mortality)” and no language restrictions. 294 abstracts were retrieved. After screening by abstract, 42 potentially relevant publications remained. Of these, 12 studies directly assessed the association of severe mental illness with mortality outcomes in individuals with schizophrenia and specifically provided detail on mortality outcomes in severe mental illness by racial or ethnic group. Only one study presented findings relating to cause-specific mortality, and most of the studies focused on suicide or all-cause mortality. With the exception of two studies, most had few individuals from ethnic minority groups, affecting the ability to report on associations for these populations. No adequately powered studies have been done outside the USA that assessed all-cause, natural-cause, and unnatural-cause mortality in ethnic minority groups with severe mental illness.

**Added value of this study**

Our findings suggest that people with severe mental illness, irrespective of ethnicity, have excess mortality compared with the general population. This observation was evident for all mortality outcomes, including suicide mortality, non-suicide unnatural-cause mortality, respiratory mortality, cardiovascular mortality, and cancer mortality. However, among people with severe mental illness, those from ethnic minority groups (black African, black Caribbean, and south Asian) had lower mortality than the reference white British population. Unlike previous work, we adjusted for the possibility that first-generation migrants might return to their country of origin prior to death (which could erroneously give the impression of so-called healthy migrant effects), since we assessed the effect of emigrations out of the cohort as a competing risk in sensitivity analyses. However, despite taking this possibility into account, observed differences in mortality persisted.

**Implications of all the available evidence**

Excess mortality is a concern in all people with severe mental illness irrespective of ethnicity. The reduced mortality in black African, black Caribbean, and south Asian groups relative to the white British group in this UK-based population with severe mental illness deserves further investigation. Our findings are consistent with results from a US study of mortality outcomes in schizophrenia, which also indicated reduced mortality in black, Hispanic, and other ethnic groups compared with non-Hispanic white Americans for most causes of death, excluding unnatural causes. These findings might indicate differential factors that might have relevance in improving mortality outcomes in people with severe mental illness.

Mental health teams within SLaM are required to assign psychiatric diagnoses according to ICD-10 for all patients.^6^, ^8^ Searches for diagnoses were done within structured fields and supplemented by a natural language-processing application developed with Generalised Architecture for Text Engineering^9^ to identify diagnosis of mental disorders according to ICD-10 in case notes and correspondence, including schizophrenia spectrum disorders (F2*) and bipolar disorders (F30 and F31). We included individuals older than age 15 years at the time of diagnosis who had any contact with SLaM services (including inpatient, outpatient, or emergency department contacts). Individuals with comorbid dementia before their diagnosis of severe mental illness were excluded. The observation period for the study was from Jan 1, 2007, to Dec 31, 2014. At-risk periods in the study were from the date of severe mental illness diagnosis to the date of death or emigration (whichever came first) or to the censor date (Dec 31, 2014) for individuals who were still alive.

Permission to conduct secondary analysis of the Clinical Record Interactive Search system was granted by the Oxfordshire Research Ethics Committee C (reference 08/H0606/71+5). Separate approvals to examine linked mortality data with approved researcher status were obtained from the UK Health & Social Care Information Centre.

### Measures

Information on mortality for the general population of England and Wales was linked to the cohort using data from the UK Office for National Statistics with the National Health Service (NHS) number, which is a unique patient identifier for all NHS health records within the UK. The NHS unique patient identifier was also used to link to all records relating to emigrations and so-called cancelled ciphers. The cancelled cipher code is ascribed to individuals who have not consulted with a general practitioner within a 3 year period if younger than 75 years or within a 1 year period if aged 75 years or above.^10^, ^11^ Individuals are sent a letter to the last known postal address and if they do not respond within 6 months their registration is cancelled.^10^ Methodological work undertaken by the Office of National Statistics using deregistration data from the Longitudinal Study indicates that health authority deregistrations are a reasonable proxy for emigration, with 95% of members from the study who were deregistered on or before census day in 2001 not found in the census.^10^, ^11^ Case-tracing procedures were done until the end of the observation period. Causes of death on death certificates were classified by the Office for National Statistics according to ICD-10^8^ and were grouped into all-cause mortality (A00–R99; U00–Y89), natural-cause mortality (A00–Q99), unnatural or external causes of mortality (U509, V01–Y89), and deaths not elsewhere classified (R00–R99). Cause-specific mortality was further categorised to include deaths from respiratory diseases (J00–J99), circulatory diseases (I00–I99), and cancers (C00–D48). Unnatural-cause mortality included deaths from suicide (X60–X84 and Y10–Y34), intentional self-harm (X60–X84), and events of undetermined intent (Y10–Y34). Unnatural-cause mortality that was not classified as suicide, self-harm, or events of undetermined intent was classified as deaths from accidents or external causes.

We included the following demographic indicators: date of birth, sex, and marital status. Postcodes of participants were linked to area-level indicators of deprivation (Index of Multiple Deprivation) at the level of Lower Super Output Area,^12^ which comprises areas with a mean of roughly 1500 residents. The Index of Multiple Deprivation takes into account deprivation across multiple domains—income, employment, health, education, barriers to housing and services, living environment, and crime—with specific weightings.^12^

Self-ascribed ethnicity was classified according to the following categories defined by the Office for National Statistics: white British, Irish, black Caribbean, and black African. Indian, Pakistani, and Bangladeshi individuals were classed into one group (south Asians) because the number of individuals was too small for separate analyses. The ICD-10 diagnostic codes F30 and F31 were categorised as affective disorders, and all other F2* diagnoses were coded as non-affective disorders. Individuals who also had a clinician-ascribed diagnosis of ICD-10 code F10–F19 (“mental and behavioural disorders due to psychoactive substance use”) were classified as having comorbid alcohol or substance misuse.^8^

### Statistical analysis

Deaths by cause and ethnicity in the cohort over the observation period were indirectly standardised by age and sex to their counterparts (resident population and deaths) from England and Wales for 2011 (mid-point of the observation period) to derive standardised mortality ratios (SMRs) with 95% CIs. For the purposes of standardisation, age was determined as the mid-point of the observation period (Jan 1, 2011) or the date of diagnosis of mental disorder if the diagnosis occurred after the mid-point. We categorised age into 10 year bands (15–24, 25–34, 35–44, 45–54, 55–64, 65–74, 75–84, and ≥85 years) corresponding to the reference population age groups. Because mortality of the standard population was given for 1 year but our target population was observed for 8 years, weights to account for the length of follow-up were derived by taking the mean observation period contributed by individuals within each corresponding age and sex band in the cohort. Each weight was then multiplied by the number of deaths recorded in each corresponding band for the standard population to provide an estimation of the expected number of deaths in the observation period.

We used Cox proportional hazards regression to estimate crude and adjusted hazard ratios (HRs) for the association of ethnicity and other covariates (sex, diagnosis, marital status, comorbid alcohol or substance misuse, and area-level deprivation) with mortality. Lexis expansion was used to derive the time-varying covariates of age (15–44, 45–64, and ≥65 years) and time since diagnosis (0–3, 3–7, and >7 years). Proportional hazards assumptions were checked by assessing interactions with survival time, examining Schoenfeld residual plots, and testing for a zero-slope in scaled residuals.^13^ Likelihood ratio tests were used to assess statistical interactions.

To assess associations with natural-cause and unnatural-cause mortality outcomes, we used a modified Cox regression approach taking into account competing risks.^14^ These models take into account the likelihood of the competing event occurring (events that remove study participants from being at risk of the event of interest—eg, through death from another cause).^14^ We first assessed the association of independent variables with natural-cause mortality, competing with unnatural-cause mortality risks. We next assessed the association of independent variables with unnatural-cause mortality, competing with natural-cause mortality risk. Sub-HRs with 95% CIs based on robust SE estimations were generated. Wald tests were used for hypothesis testing.

First-generation ethnic minorities might migrate back to their country of origin when they are unwell or before death, which might lead to a biased under-estimation of mortality risk through numerator–denominator mismatch.^15^ Therefore, we did a sensitivity analysis to re-assess associations between ethnicity and all-cause mortality by using competing-risks regression, specifying emigration out of the cohort as a competing event as opposed to a censored event.

Statistical analyses were done in Stata, version 12.

### Role of the funding source

The funders of the study had no role in study design, data collection, data analysis, data interpretation, writing of the report, or the decision to submit for publication. The corresponding author had full access to all the data in the study and had final responsibility for the decision to submit for publication.

## Results

After excluding individuals with incomplete data and those from ethnic groups other than the ones studied here, 18 201 were included in the cohort, contributing a median follow-up of 6·36 years (IQR 3·26–9·92) and a total of 1767 deaths by the study censor date (Dec 31, 2014; figure). 5041 (27·7%) individuals had an affective diagnosis at baseline and roughly half of the cohort belonged to an ethnic minority group (table 1).

The main causes of death were from circulatory disease (including cardiovascular and cerebrovascular disease; 474 [27%] deaths), cancers (308 [17%]), respiratory disease (290 [16%]), and external causes (including suicides and non-suicide causes; 189 [11%]). Irrespective of ethnicity, SMR was elevated for all causes of death in people with severe mental illness (2·67, 95% CI 2·56–2·78; table 2). SMRs for cardiovascular and respiratory mortality were increased by 2–4 times for almost all ethnic groups and suicide by 5–10 times in all ethnic groups. SMRs for cancer mortality were also raised, albeit to a lower extent (1·45, 1·32–1·60), across the entire cohort, but south Asian people with severe mental illness had a reduced SMR for cancer mortality (0·49, 0·21–0·96; table 2). SMRs for deaths due to circulatory causes were elevated in black African people with severe mental illness (3·85, 2·71–5·31) relative to the white British group with severe mental illness (2·66, 2·38–2·96), albeit with overlapping 95% CIs.

In Cox proportional hazards regression models using the white British group as reference, there was evidence of non-proportional hazards in the association of ethnicity with all-cause mortality (likelihood ratio test for interaction of ethnicity with time p=0·0055; χ^2^ test for non-zero slope in Schoenfeld residuals p=0·013; appendix pp 1–2). Proportional hazards assumptions were met when an interaction of time with ethnicity was fitted (table 3). Overall, relative to white British people with severe mental illness, mortality was reduced in black African, black Caribbean, and south Asian individuals with severe mental illness (likelihood ratio test for interaction with time since diagnosis p<0·0001); this difference reduced slightly for the black Caribbean group as time since diagnosis increased (table 3). Female sex and affective diagnoses (rather than non-affective diagnoses) were associated with a lower all-cause mortality, whereas comorbid alcohol or substance misuse and being single, divorced, widowed, or separated were associated with a 19% increased risk of all-cause mortality after adjustment for all other variables (table 4).

In competing risks regression models, women had a reduced risk of unnatural-cause mortality relative to men (table 5). Affective psychosis was associated with a reduced natural-cause mortality, but no difference by diagnosis was found for unnatural-cause mortality (table 5). Comorbid alcohol or substance misuse was associated with a doubling in unnatural-cause mortality (table 5). With the exception of the Irish group for natural-cause mortality, mortality was reduced for each of the ethnic minority groups (relative to white British people with severe mental illness) for both natural-cause and unnatural-cause mortality in adjusted models (table 5). In sensitivity analyses with emigrations out of the cohort as a competing risk, sub-HR estimates for ethnicity and all-cause mortality followed similar trends to those highlighted in table 3 (appendix p 3)—ie, the observed differences in mortality were not affected by emigration.

In a post-hoc analysis, we fitted an interaction between ethnicity and severe mental illness diagnoses (affective *vs* non-affective) in their association with all-cause mortality to assess the possibility that the association of ethnicity with all-cause mortality might be modified by diagnosis. We did not find any evidence in support of effect modification by ethnicity in these models (likelihood ratio test for interaction p=0·39; appendix p 4).

## Discussion

Findings from our cohort study confirm that people with severe mental illness have an elevated mortality risk compared with the general population. Mortality from respiratory disorders and cardiovascular disease was elevated by up to 4 times and suicide by 5–10 times across almost all ethnic minority groups.

Among individuals with severe mental illness, black African and black Caribbean people had reduced mortality compared with white British people for all causes, natural causes, and unnatural causes of deaths. Similar trends, particularly for natural-cause mortality, were observed in the south Asian group. As time since diagnosis increased, the HRs for all-cause mortality between black Caribbean and white British individuals with severe mental illness became more similar, but the black Caribbean group continued to have a reduced mortality risk relative to the white British group by the end of the study.

Other associations with mortality in our study are consistent with the wider literature; in particular, comorbid alcohol and substance misuse diagnoses were associated with a doubling in risk of unnatural-cause mortality,^16^ female sex was associated with reduced all-cause and unnatural-cause mortality relative to men,^16^, ^17^ affective diagnoses were associated with a reduced risk of natural-cause mortality relative to non-affective diagnoses,^18^ and being single, divorced, widowed, or separated was associated with an increased mortality risk.^19^

The use of a large cohort from an ethnically diverse location allowed us to assess differences in mortality outcomes in severe mental illness for each ethnic minority group. Previous studies^16^, ^17^ did not have adequate power to detect differences by ethnicity. By prospectively assessing mortality and tracing emigrations out of the cohort, we were able to assess the possibility of bias through emigration. The long follow-up of 8 years allowed a detailed assessment. Our sample was comprehensive: since we would have included everyone in contact with the mental health trust with severe mental illness, coverage within this area was likely to have been almost complete.^6^ Although we might have excluded people with psychosis who sought private health care, this number is likely to have been very small.^6^ It is of course possible that people with non-psychotic bipolar disorders were more likely than those with psychotic disorders to access private services; however, in the UK state-funded services tend to provide most health care and use of private services tends to be minimal;^6^ therefore, this potential source of bias was likely to have been low. The findings are generalisable insofar as the location (urban inner city) typifies areas where ethnic minority communities tend to reside.

Our study had a few limitations. First, diagnoses of severe mental illness were not based on research diagnostic criteria. Racially biased diagnostic practices might have led to ethnic minority groups being more likely to receive a psychosis diagnosis that did not meet research diagnostic criteria,^20^ which might have led to a lower recorded mortality risk if such mis-diagnosis in ethnic minority groups meant that these individuals were likely to have had less severe mental illness. However, the direction of association observed in our study is consistent with findings from another study^16^ using research diagnostic criteria by clinicians blinded to the ethnicity of participants. Second, the south Asian group in our study might have masked important differences for Indian, Pakistani, and Bangladeshi individuals. Although we adjusted for area-level deprivation, indicators of individual-level socioeconomic position were not available. Additionally, future work could assess the role of mediators of premature mortality, particularly tobacco use, type 2 diabetes, obesity, and hypertension.

Presently, work is underway on this data source to develop natural language-processing applications to extract information on physical health from unstructured or free text,^7^ as well as to use database linkage with primary care records to enhance assessment of cardiovascular disease and other physical health indicators within the record.^21^ Other natural language-processing applications developed in the Clinical Record Interactive Search system in current research use include those ascertaining text on tobacco^22^ and cannabis use,^23^ medications for diabetes and other physical disorders,^7^ and more than 70 different mental health symptoms.^24^ These efforts have been supplemented by a range of new algorithms for ascertaining recorded body-mass index and mentions of comorbid physical disorders and use of several common illicit drugs. The role of these factors as potential mediators for premature mortality could thus be assessed in future work using this data source.

Overall, differences in SMRs in our cohort reflect those noted in previous work.^1^, ^4^, ^17^, ^25^ SMRs for cardiovascular and respiratory mortality were elevated irrespective of ethnicity. SMRs for cancers are also consistent with the findings from a meta-analysis of mortality outcomes in schizophrenia.^1^ Among individuals with severe mental illness, the finding of a lower all-cause SMR in ethnic minority groups relative to non-minority reference groups has been reported in a study from the USA.^4^

The finding of a much lower SMR for cancers in south Asian groups with severe mental illness is still consistent with wider findings from the literature^1^ and might also reflect a lower prevalence of cancers reported for this ethnic group compared with non-south Asians.^26^ Investigators of a US study of more than 1 million people with schizophrenia^4^ also reported lower SMR for cancer mortality in all the ethnic minority groups surveyed (black non-Hispanic [1·2, 95% CI 1·2–1·3], Hispanic [1·6, 1·4–1·7], and other non-Hispanic [1·4, 1·2–1·7]) than in white non-Hispanic individuals (2·0, 2·0–2·1). Our finding of a lower SMR for suicide in the black Caribbean group relative to the white British group with severe mental illness is broadly consistent with previous research from the UK.^27^, ^28^ Investigators of the AESOP-10 study^16^ used 10 year follow-up data from 557 people with first-episode psychosis and determined diagnoses at baseline by assessors blinded to the ethnicity of respondents. In this study,^16^ unnatural-cause mortality was lower in black and minority ethnic groups than in white British people, although 95% CIs were wide. Among people with schizophrenia, suicide risk has been reported to be lower in first-generation migrants than in native Dutch people in the Netherlands,^29^ as well as lower in black non-Hispanic people than in white non-Hispanic people in the USA.^4^

The apparently reduced mortality risk in ethnic minority groups with severe mental illness deserves further exploration. Social factors that increase mortality risk might be less prevalent in black African, black Caribbean, and south Asian groups with severe mental illness than in white British people with severe mental illness. For example, findings from previous studies have indicated the role of ethnic density (ie, a high proportion of people of the same ethnicity living in the same area) in reducing self-harm and suicide risks in ethnic minorities in the UK^30^, ^31^ and the Netherlands.^32^ Group density effects are also associated with reduced alcohol use in black Caribbean, black African, and Indian individuals living in areas of high ethnic density.^33^ Social support and buffering from discrimination and social isolation, as well as protective social norms, have been implicated in these effects.^30^, ^33^, ^34^ The catchment area for this study—similar to many other urban locations where ethnic minority communities live—is notable for being ethnically diverse and having one of the largest black Caribbean and black African communities in the UK.^5^, ^7^ Therefore, these socioenvironmental factors might have played a part in mediating mortality risk for our study cohort. By contrast, an increasing body of evidence shows an elevated cardiovascular risk (eg, type 2 diabetes) in these groups,^2^ and these complex interactions will need to be explored in future research.^35^

Other factors might also have led to the lower mortality risk in black Caribbean, black African, and south Asian individuals with severe mental illness, relative to the white British group with severe mental illness. For example, if mortality rates were higher in these ethnic minority groups before receiving a diagnosis for a severe mental illness (and therefore their entry into the study cohort), this could have led to an artificially reduced risk of mortality relative to the white British group. Another possibility is that people who made first contact with services through either primary care or emergency departments might have had their physical health optimised, leading to improved mortality outcomes. However, findings from studies assessing ethnic minority pathways into care^36^, ^37^ have consistently indicated that ethnic minority groups with psychosis are less likely than white British individuals to make first contact through these routes—in particular, black individuals with psychosis are more likely to experience criminal justice routes into care—so this possibility seems less likely but could be explored in future work. Finally, other factors such as differences in physical health (eg, presence of type 2 diabetes^38^ and other cardiovascular disease indicators), differential prescribing of psychotropic medications by ethnicity (including antipsychotic medications and associated weight gain), and the presence of untreated primary health conditions (including cardiovascular disease)^21^ might have had a role in the differential mortality risk by ethnicity observed, and should be investigated in future work.

The catchment area for the study reflects relatively recent migration trends spanning one to two generations. Therefore, a further possibility is that the trends in our study reflect migrant groups being selectively more resilient to ill-health effects. Lower suicide risk in some ethnic minority groups, with protective effects being lost in younger and second-generation migrants, has previously been noted,^27^, ^29^ leading some to implicate healthy migrant effects or the loss of acculturation health benefits over time and generation.^27^ Our findings extend these observations to natural-cause mortality in severe mental illness. We were unable to assess the effect of migrant or generational status, but this alongside the role of acculturation should be reviewed in future research.

Irrespective of ethnicity, people with severe mental illness have excess mortality, underlining an urgent need to address tractable causes within this group of people. Reduced mortality risk in black African, black Caribbean, and south Asian groups with severe mental illness, relative to a white British reference group with severe mental illness, might be due to several factors, including but not limited to differential socioenvironmental factors, differences in underlying physical health, and differences in the prescribing of psychotropic medications. These factors could be relevant to improving mortality outcomes in all people with severe mental illness.

## Figures and Tables

**Figure:**
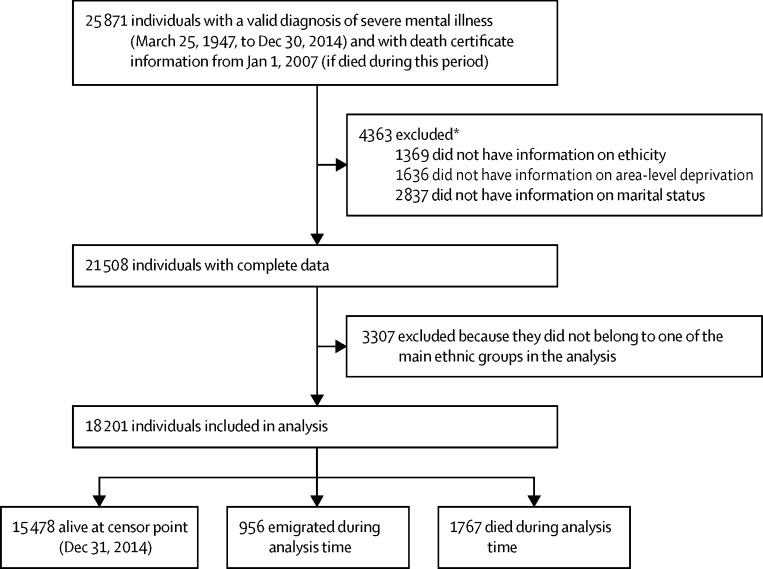
Study flow for regression models *Some individuals had missing information in more than one category.

**Table 1:** Baseline characteristics and crude mortality rates

			**n (%)**	**All causes**		**Natural causes**		**Unnatural causes**	
				Number of deaths	Crude mortality per 100 000 person years (95% CI)	Number of deaths	Crude mortality per 100 000 person years (95% CI)	Number of deaths	Crude mortality per 100 000 person years (95% CI)

Total cohort			18 201	1767	1439·7 (1374·1–1508·4)	1417	1154·6 (1096·0–1216·3)	192	156·4 (135·8–180·2)
Diagnosis									
	Affective		5041 (27·7%)	409	1338·9 (1215·3–1475·2)	327	1070·5 (960·5–1193·0)	51	167·0 (126·9–219·7)
	Non-affective		13 160 (72·3%)	1358	1473·1 (1396·8–1553·6)	1090	1182·4 (1114·3–1254·7)	141	153·0 (129·7–180·4)
Ethnicity									
	White British		9047 (49·7%)	1130	1989·8 (1877·1–2109·2)	913	1607·7 (1506·7–1715·4)	125	220·1 (184·7–262·3)
	Black African		2510 (13·8%)	106	645·9 (533·9–781·3)	82	499·7 (402·42–620·4)	18	109·7 (69·1–174·1)
	Black Caribbean		4840 (26·6%)	332	880·5 (790·7–980·5)	256	678·9 (600·7–767·4)	33	87·5 (62·2–123·1)
	South Asian		1256 (6·9%)	95	1178·2 (963·6–1440·6)	77	955·0 (763·8–1194·0)	11	136·4 (75·6–246·3)
	Irish		548 (3·0%)	104	2765·9 (2282·3–3352·1)	89	2367·0 (1923·0–2913·6)	5	133·0 (55·3–319·5)
Sex									
	Male		9610 (52·8%)	908	1340·3 (1255·9–1430·4)	707	1043·6 (969·4–1123·4)	132	194·8 (164·3–231·1)
	Female		8591 (47·2%)	859	1562·3 (1461·2–1670·3)	710	1291·3 (1199·7–1389·8)	60	109·1 (84·7–140·5)
Area-level deprivation (IMD rank)									
	Median (IQR)		7200 (4737–11 610)*	··	··	··	··	··	··
	IMD fifths								
		First (least deprived)	3667 (20·2%)	377	1442·3 (1303·8–1595·5)	305	1116·8 (1043·0–1305·4)	33	126·2 (89·8–177·6)
		Second	3646 (20·0%)	342	1332·4 (1198·4–1481·4)	273	1063·6 (944·6–1197·5)	35	136·4 (97·9–189·9)
		Third	3629 (19·9%)	313	1250·5 (1119·3–1397·0)	247	986·8 (871·1–117·9)	37	147·8 (107·1–204·0)
		Fourth	3673 (20·2%)	371	1493·2 (1348·7–1653·2)	297	1195·4 (1066·9–1339·4)	39	156·9 (114·7–214·8)
		Fifth (most deprived)	3586 (19·7%)	364	1729·4 (1560·5–1916·5)	295	1401·5 (1250·4–1571·0)	48	220·0 (171·9–302·6)
Marital status									
	Married or cohabiting		2781 (15·2%)	267	1583·1 (1404·2–1784·9)	220	1304·4 (1143·0–1488·7)	22	130·4 (85·9–198·1)
	Single, divorced, widowed, or separated		15 420 (84·7%)	1500	1416·9 (1347·0–1490·4)	1197	1130·7 (1068·4–1196·6)	170	160·6 (138·2–186·6)
Comorbid substance misuse diagnosis									
	None		15 046 (82·7%)	1519	1505·8 (1431·9–1583·4)	1251	1240·1 (1173·3–1310·8)	128	126·9 (106·7–150·9)
	Present		3155 (17·3%)	248	1134·8 (1002·0–1285·2)	166	759·6 (652·4–884·4)	64	292·9 (229·2–374·2)

IMD=Index of Multiple Deprivation. Data for 158 individuals who died from unknown causes not elsewhere classified (R00–99) have not been displayed.

* Range for the IMD at the level of Lower Super Output Area for England is 1 (most deprived) to 32 844 (least deprived); therefore, the rank of 7200 is out of 32 844 small areas in England—ie, the study area is among the 22% (IQR 14–35) most deprived in the country.

**Table 2:** SMRs for selected causes in people with severe mental illness

		**Total sample**	**White British**	**Black Caribbean**	**Black African**	**South Asian**	**Irish**
**All-cause mortality**							
Number of deaths		2390	1317	384	127	106	115
SMR (95% CI)		2·67 (2·56–2·78)	2·96 (2·80–3·12)	2·11 (1·90–2·33)	2·98 (2·48–3·54)	2·34 (1·91–2·83)	2·59 (2·14–3·11)
p value		<0·001	<0·001	<0·001	<0·001	<0·001	<0·001
**Natural-cause mortality**							
Circulatory system, including cardiovascular and cerebrovascular disease							
	Number of deaths	647	331	121	37	28	33
	SMR (95% CI)	2·65 (2·45–2·86)	2·66 (2·38–2·96)	2·46 (2·04–2·94)	3·85 (2·71–5·31)	2·33 (1·55–3·37)	2·58 (1·78–3·63)
	p value	<0·001	<0·001	<0·001	<0·001	<0·001	0·021
Respiratory system, including COPD and pneumonia							
	Number of deaths	370	237	29	11	23	24
	SMR (95% CI)	3·38 (3·04–3·74)	4·19 (3·67–4·76)	1·32 (0·89–1·90)	2·87 (1·43–5·14)	4·38 (2·78–6·57)	4·06 (2·60–6·04)
	p value	<0·001	<0·001	0·17	0·0043	<0·001	<0·001
Cancers							
	Number of deaths	433	244	63	43	≤10*	18
	SMR (95% CI)	1·45 (1·32–1·60)	1·68 (1·47–1·90)	1·05 (0·80–1·34)	1·79 (1·19–2·59)	0·49 (0·21–0·96)	1·25 (0·74–1·97)
	p value	<0·001	<0·001	0·76	0·0062	0·035	0·41
**Unnatural-cause mortality**							
Suicide, self-harm, and events of undetermined intent							
	Number of deaths	138	71	21	14	≤10*	≤10*
	SMR (95% CI)	7·65 (6·43–9·04)	10·41 (8·13–13·13)	4·87 (3·01–7·44)	6·71 (3·67–11·26)	7·41 (2·98–15·27)	6·67 (1·38–19·49)
	p value	<0·001	<0·001	<0·001	<0·001	<0·001	0·024
Other external causes of mortality†							
	Number of deaths	126	77	19	≤10*	≤10*	≤10*
	SMR (95% CI)	4·01 (3·34–4·78)	5·79 (4·57–7·23)	2·68 (1·61–4·19)	1·42 (0·39–3·63)	1·58 (1·03–7·38)	2·78 (0·57–8·13)
	p value	<0·001	<0·001	<0·001	0·62	0·047	0·19

SMRs standardised by age and sex to the population of England and Wales in 2011. Total number of observed deaths is greater than in regression models, as mortality in the sample was assessed irrespective of missing data for covariates and ethnicity. p values derived through Byaar approximation. SMR=standardised mortality ratio. COPD=chronic obstructive pulmonary disease.

* Exact number not shown to protect patient confidentiality.

† Included accidents, falls, and assaults.

**Table 3:** Adjusted HRs for all-cause mortality, by ethnic group and time since diagnosis

	**Total cases**	**Number of deaths**	**Time since diagnosis**											
			0 to <3·21 years				3·21 to <6·71 years				≥6·71 years			
			Crude		Adjusted*		Crude		Adjusted*		Crude		Adjusted*	
			HR (95% CI)	p value	HR (95% CI)	p value	HR (95% CI)	p value	HR (95% CI)	p value	HR (95% CI)	p value	HR (95% CI)	p value
White British	9047	1130	1	··	1	··	1	··	1	··	1	··	1	··
Black Caribbean	4840	332	0·27 (0·21–0·36)	<0·001	0·35 (0·26–0·46)	<0·0001	0·46 (0·37–0·56)	<0·001	0·58 (0·47–0·72)	<0·0001	0·48 (0·40–0·58)	<0·0001	0·67 (0·56–0·81)	<0·0001
Black African	2510	106	0·36 (0·26–0·50)	<0·001	0·65 (0·46–0·91)	0·013	0·37 (0·27–0·51)	<0·0001	0·68 (0·49–0·94)	0·021	0·24 (0·16–0·35)	<0·0001	0·43 (0·30–0·64)	<0·0001
South Asian	1256	95	0·59 (0·41–0·86)	0·0060	0·69 (0·48–1·01)	0·055	0·58 (0·40–0·83)	0·003	0·69 (0·48–0·99)	0·042	0·59 (0·41–0·84)	0·0040	0·73 (0·51–1·04)	0·086
Irish	548	104	1·40 (0·97–2·00)	0·069	0·93 (0·65–1·34)	0·71	1·52 (1·09–2·12)	0·014	1·03 (0·74–1·44)	0·85	1·17 (0·82–1·66)	0·38	0·88 (0·62–1·25)	0·48

Models derived from Cox regression analyses; p values calculated with the Wald test. HR=hazard ratio.

* Adjusted for age, sex, diagnosis, marital status, area-level deprivation, and comorbid alcohol or substance misuse.

**Table 4:** Adjusted HRs for all-cause mortality, by covariates

	**Total cases**	**Number of deaths**	**Crude estimates**				Adjusted estimates*	
			HR (95% CI)	p value (Wald test)	p value (LRT)	HR (95% CI)	p value (Wald test)	p value (LRT)
**Sex**								
Male	9610	908	1	··	0·00020	1	··	0·0030
Female	8591	859	1·19 (1·09–1·31)	<0·0001	··	0·86 (0·79–0·95)	0·0030	··
**Diagnosis**								
Non-affective	13 160	1358	1	··	0·28	1	··	0·0033
Affective	5041	409	0·94 (0·84–1·05)	0·28	··	0·84 (0·75–0·95)	0·0040	··
**Marital status**								
Married or cohabiting	2781	267	1	··	0·031	1	··	0·0087
Single, divorced, widowed, or separated	15 420	1500	0·86 (0·76–0·98)	0·028	··	1·19 (1·04–1·36)	0·010	··
**Comorbid alcohol or substance misuse**								
None	15 046	1519	1	··	<0·001	1	··	0·016
Present	3155	248	0·75 (0·65–0·85)	<0·0001	··	1·19 (1·04–1·37)	0·015	··
**Area-level deprivation (IMD fifths)**								
First (least deprived)	3667	377	1	··	<0·0001	1	··	0·30
Second	3646	342	0·93 (0·80–1·07)	0·32	··	0·91 (0·78–1·05)	0·19	··
Third	3629	313	0·87 (0·75–1·01)	0·075	··	0·86 (0·74–1·00)	0·054	··
Fourth	3673	371	1·05 (0·91–1·22)	0·48	··	0·93 (0·81–1·08)	0·36	··
Fifth (most deprived)	3586	364	1·26 (1·09–1·46)	0·0010	··	0·98 (0·85–1·14)	0·80	··

Models derived from Cox regression analyses. HR=hazard ratio. LRT=likelihood ratio test. IMD=Index of Multiple Deprivation.

* Adjusted for age, ethnicity, and all other variables shown in the table.

**Table 5:** Sub-HRs for natural-cause and unnatural-cause mortality

	**Natural causes**							**Unnatural causes**						
	Number of deaths	Crude estimates			Adjusted estimates*			Number of deaths	Crude estimates			Adjusted estimates*		
		Sub-HR (95% CI)	p value (Wald test)	p value (LRT)	Sub-HR (95% CI)	p value (Wald test)	p value (LRT)		Sub-HR (95% CI)	p value (Wald test)	p value (LRT)	Sub-HR (95% CI)	p value (Wald test)	p value (LRT)
**Ethnicity**														
White British	913	1	··	<0·0001	1	··	<0·0001	125	1	··	0·00010	1	··	0·00030
Black African	82	0·31 (0·25–0·39)	<0·0001	··	0·49 (0·39–0·61)	<0·0001	··	18	0·52 (0·32–0·85)	0·009	··	0·55 (0·33–0·92)	0·023	··
Black Caribbean	256	0·42 (0·37–0·48)	<0·0001	··	0·52 (0·45–0·59)	<0·0001	··	33	0·43 (0·30–0·64)	<0·0001	··	0·42 (0·28–0·63)	<0·0001	··
South Asian	77	0·59 (0·47–0·74)	<0·0001	··	0·66 (0·53–0·83)	<0·0001	··	11	0·64 (0·34–1·18)	0·15	··	0·68 (0·36–1·28)	0·23	··
Irish	89	1·47 (1·18–1·83)	0·0010	··	1·08 (0·87–1·34)	0·48	··	5	0·60 (0·25–1·48)	0·27	··	0·59 (0·24–1·46)	0·26	··
**Sex**														
Male	707	1	··	<0·0001	1	··	0·13	132	1	··	0·00010	1	··	0·0020
Female	710	1·25 (1·12–1·38)	<0·0001	··	0·92 (0·83–1·02)	0·13	··	60	0·54 (0·40–0·73)	<0·0001	··	0·61 (0·44–0·83)	0·0020	··
**Diagnosis**														
Non-affective	327	1	··	0·11	1	··	0·00040	51	1	··	0·75	1	··	0·98
Affective	1090	0·90 (0·80–1·02)	0·11	··	0·80 (0·70–0·90)	<0·0001	··	141	1·05 (0·77–1·45)	0·75	··	1·00 (0·72–1·39)	0·98	··
**Marital status**														
Married or cohabiting	220	1	··	0·050	1	··	0·036	22	1	··	0·26	1	··	0·64
Single, divorced, widowed, or separated	1197	0·87 (0·75–1·00)	0·050	··	1·17 (1·01–1·36)	0·036	··	170	1·29 (0·83–2·01)	0·26	··	1·12 (0·71–1·76)	0·64	··
**Comorbid alcohol or substance misuse**														
None	1251	1	··	<0·0001	1	··	0·12	128	1	··	<0·0001	1	··	<0·0001
Present	166	0·61 (0·51–0·71)	<0·0001	··	0·88 (0·74–1·04)	0·12	··	64	2·36 (1·75–3·19)	<0·0001	··	2·08 (1·53–2·83)	<0·0001	··
**Area-level deprivation (IMD fifths)**														
First (least deprived)	305	1	··	0·0010	1	··	0·16	33	1	··	0·13	1	··	0·24
Second	273	0·91 (0·77–1·07)	0·27	··	0·87 (0·75–1·02)	0·086	··	35	1·08 (0·67–1·74)	0·75	··	1·03 (0·64–1·66)	0·89	··
Third	247	0·85 (0·72–1·00)	0·054	··	0·91 (0·78–1·07)	0·014	··	37	1·18 (0·74–1·88)	0·50	··	1·17 (0·73–1·87)	0·52	··
Fourth	297	1·03 (0·88–1·21)	0·73	··	0·91 (0·78–1·07)	0·25	··	39	1·23 (0·77–1·96)	0·38	··	1·20 (0·75–1·91)	0·44	··
Fifth (most deprived)	295	1·19 (1·02–1·40)	0·029	··	0·91 (0·77–1·06)	0·23	··	48	1·70 (1·09–2·64)	0·019	··	1·60 (1·02–2·52)	0·04	··

Models took into account competing risks. Data for 158 individuals who died from unknown causes not elsewhere classified (R00–99) have not been displayed. HR=hazard ratio. LRT=likelihood ratio test. IMD=Index of Multiple Deprivation.

* Adjusted for age and all other variables shown in the table.

## References

[1] Saha S, Chant D, McGrath J (2007). A systematic review of mortality in schizophrenia: is the differential mortality gap worsening over time?. Arch Gen Psychiatry.

[2] Ward M, Druss B (2015). The epidemiology of diabetes in psychotic disorders. Lancet Psychiatry.

[3] DE Hert M, Correll CU, Bobes J (2011). Physical illness in patients with severe mental disorders. I. Prevalence, impact of medications and disparities in health care. World Psychiatry.

[4] Olfson M, Gerhard T, Huang C, Crystal S, Stroup TS (2015). Premature mortality among adults with schizophrenia in the United States. JAMA Psychiatry.

[5] Das-Munshi J, Ashworth M, Gaughran F (2016). Ethnicity and cardiovascular health inequalities in people with severe mental illnesses: protocol for the E-CHASM study. Soc Psychiatry Psychiatr Epidemiol.

[6] Stewart R, Soremekun M, Perera G (2009). The South London and Maudsley NHS Foundation Trust Biomedical Research Centre (SLAM BRC; case register: development and descriptive data. BMC Psychiatry.

[7] Perera G, Broadbent M, Callard F (2016). Cohort profile of the South London and Maudsley NHS Foundation Trust Biomedical Research Centre (SLaM BRC; Case Register: current status and recent enhancement of an Electronic Mental Health Record-derived data resource. BMJ Open.

[8] WHO (1992). The ICD-10 classification of mental and behavioural disorders: clinical descriptions and diagnostic guidelines.

[9] Cunningham H, Tablan V, Roberts A, Bontcheva K (2013). Getting more out of biomedical documents with GATE's full lifecycle open source text analytics. PLoS Comput Biol.

[10] Johnson B, Blackwell L (2007). Review of methods for estimating life expectancy by social class using the ONS Longitudinal Study. Health Stat Q.

[11] Office for National Statistics (2007). Health statistics quaterly, no. 35.

[12] Noble M, Wright G, Smith G, Dibben C (2006). Measuring multiple deprivation at the small-area level. Environ Plan A.

[13] Schoenfeld D (1982). Partial residuals for the proportional hazards regression model. Biometrika.

[14] Fine JP, Gray RJ (1999). A proportional hazards model for the subdistribution of a competing risk. J Am Stat Assoc.

[15] Razum O (2006). Commentary: of salmon and time travellers—musing on the mystery of migrant mortality. Int J Epidemiol.

[16] Reininghaus U, Dutta R, Dazzan P (2014). Mortality in schizophrenia and other psychoses: a 10-year follow-up of the ÆSOP first-episode cohort. Schizophr Bull.

[17] Dutta R, Murray RM, Allardyce J, Jones PB, Boydell JE (2012). Mortality in first-contact psychosis patients in the UK: a cohort study. Psychol Med.

[18] Khan A, Faucett J, Morrison S, Brown WA (2013). Comparative mortality risk in adult patients with schizophrenia, depression, bipolar disorder, anxiety disorders, and attention-deficit/hyperactivity disorder participating in psychopharmacology clinical trials. JAMA Psychiatry.

[19] Hamer M, Stamatakis E, Steptoe A (2008). Psychiatric hospital admissions, behavioral risk factors, and all-cause mortality: the scottish health survey. Arch Intern Med.

[20] Rockett IR, Lian Y, Stack S, Ducatman AM, Wang S (2009). Discrepant comorbidity between minority and white suicides: A national multiple cause-of-death analysis. BMC Psychiatry.

[21] Woodhead C, Ashworth M, Broadbent M (2016). Cardiovascular disease treatment among patients with severe mental illness: a data linkage study between primary and secondary care. Br J Gen Pract.

[22] Wu CY, Chang CK, Robson D (2013). Evaluation of smoking status identification using electronic health records and open-text information in a large mental health case register. PLoS One.

[23] Patel R, Wilson R, Jackson R (2016). Association of cannabis use with hospital admission and antipsychotic treatment failure in first episode psychosis: an observational study. BMJ Open.

[24] Jackson RG, Patel R, Jayatilleke N (2017). Natural language processing to extract symptoms of severe mental illness from clinical text: the Clinical Record Interactive Search Comprehensive Data Extraction (CRIS-CODE) project. BMJ Open.

[25] Chang CK, Hayes RD, Broadbent M (2010). All-cause mortality among people with serious mental illness (SMI), substance use disorders, and depressive disorders in southeast London: a cohort study. BMC Psychiatry.

[26] Mangtani P, Maringe C, Rachet B, Coleman MP, dos Santos Silva I (2010). Cancer mortality in ethnic South Asian migrants in England and Wales (1993–2003): patterns in the overall population and in first and subsequent generations. Br J Cancer.

[27] McKenzie K, van Os J, Samele C, for the UK700 Group (2003). Suicide and attempted suicide among people of Caribbean origin with psychosis living in the UK. Br J Psychiatry.

[28] McKenzie K, van Os J, Fahy T (1995). Psychosis with good prognosis in Afro-Caribbean people now living in the United Kingdom. BMJ.

[29] Termorshuizen F, Wierdsma AI, Visser E (2012). Psychosis and suicide risk by ethnic origin and history of migration in the Netherlands. Schizophr Res.

[30] Bécares L, Das-Munshi J (2013). Ethnic density, health care seeking behaviour and expected discrimination from health services among ethnic minority people in England. Health Place.

[31] Neeleman J, Wessely S (1999). Ethnic minority suicide: a small area geographical study in south London. Psychol Med.

[32] Termorshuizen F, Braam AW, van Ameijden EJ (2015). Neighborhood ethnic density and suicide risk among different migrant groups in the four big cities in the Netherlands. Soc Psychiatry Psychiatr Epidemiol.

[33] Bécares L, Nazroo J, Stafford M (2011). The ethnic density effect on alcohol use among ethnic minority people in the UK. J Epidemiol Community Health.

[34] Das-Munshi J, Bécares L, Boydell JE (2012). Ethnic density as a buffer for psychotic experiences: findings from a national survey (EMPIRIC). Br J Psychiatry.

[35] Das-Munshi J, Stewart R, Morgan C, Nazroo J, Thornicroft G, Prince M (2016). Reviving the ‘double jeopardy’ hypothesis: physical health inequalities, ethnicity and severe mental illness. Br J Psychiatry.

[36] Bhui K, Ullrich S, Coid JW (2014). Which pathways to psychiatric care lead to earlier treatment and a shorter duration of first-episode psychosis?. BMC Psychiatry.

[37] Anderson KK, Flora N, Archie S, Morgan C, McKenzie K (2014). A meta-analysis of ethnic differences in pathways to care at the first episode of psychosis. Acta Psychiatr Scand.

[38] Das-Munshi J, Ashworth M, Dewey ME (2016). Type 2 diabetes mellitus in people with severe mental illness: inequalities by ethnicity and age. Cross-sectional analysis of 588 408 records from the UK. Diabet Med.

