# Supplementary material for: Ethnicity and excess mortality in severe mental illness: a cohort study
Source: Lancet Psychiatry. 2017 May;4(5):389–99. doi: 10.1016/S2215-0366(17)30097-4 (PMC5406616; doi:10.1016/S2215-0366(17)30097-4)
Supplement: Supplementary appendix [file mmc1.pdf]

THE LANCET
Psychiatry

Supplementary appendix

This appendix formed part of the original submission and has been peer reviewed. We post it as supplied by the authors.

Supplement to: Das-Munshi J, Chang C-K, Dutta R, et al. Ethnicity and excess mortality in severe mental illness: a cohort study. *Lancet Psychiatry* 2017; published online March 16. http://dx.doi.org/10.1016/S2215-0366(17)30097-4.

Supplementary material

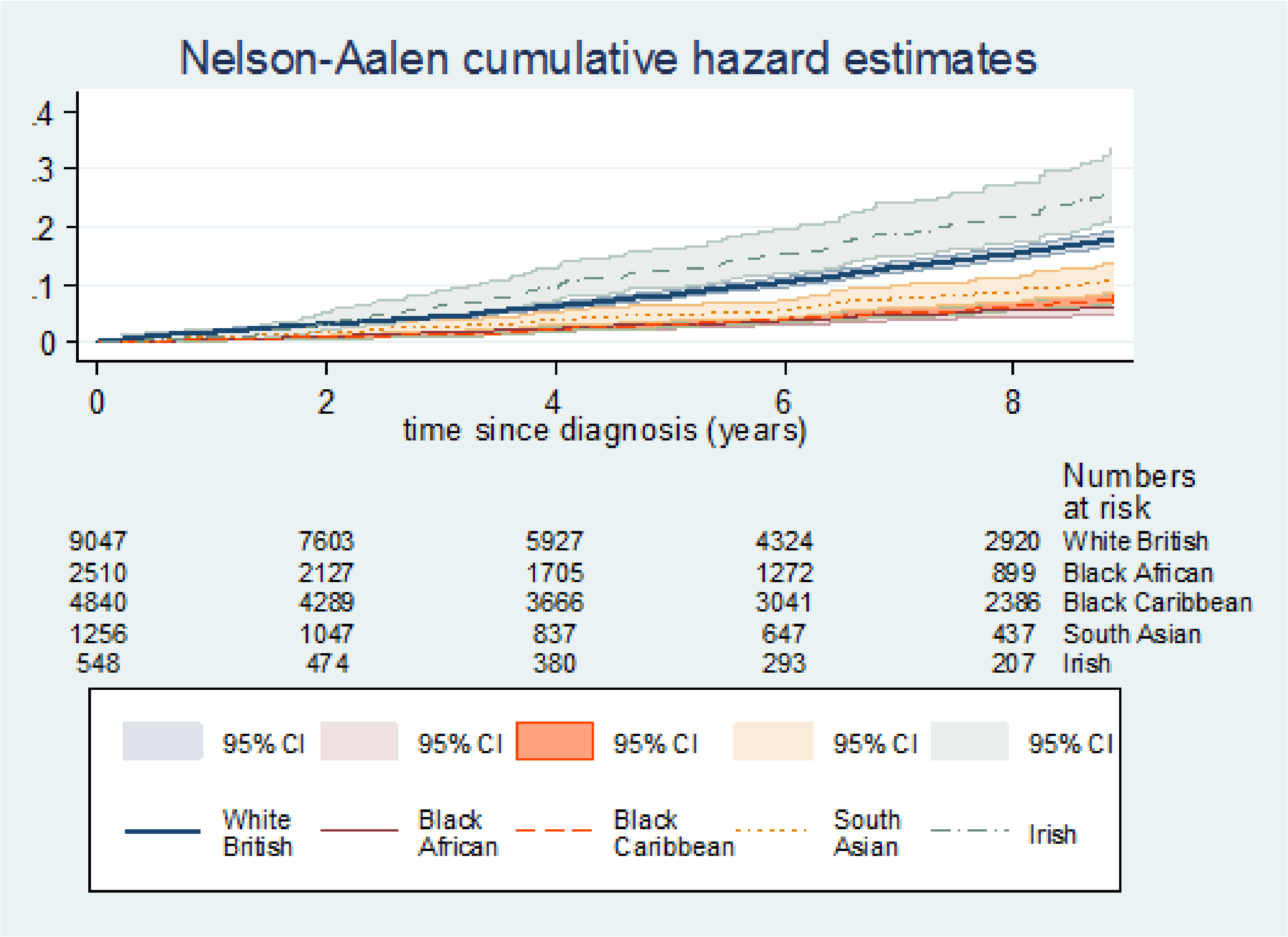

Figure 1 Cumulative hazard estimates for all-cause mortality by ethnic group, by time since SMI diagnosis

Supplementary material

Figure 2 Cumulative hazard estimates for all-cause mortality by ethnic group, by age

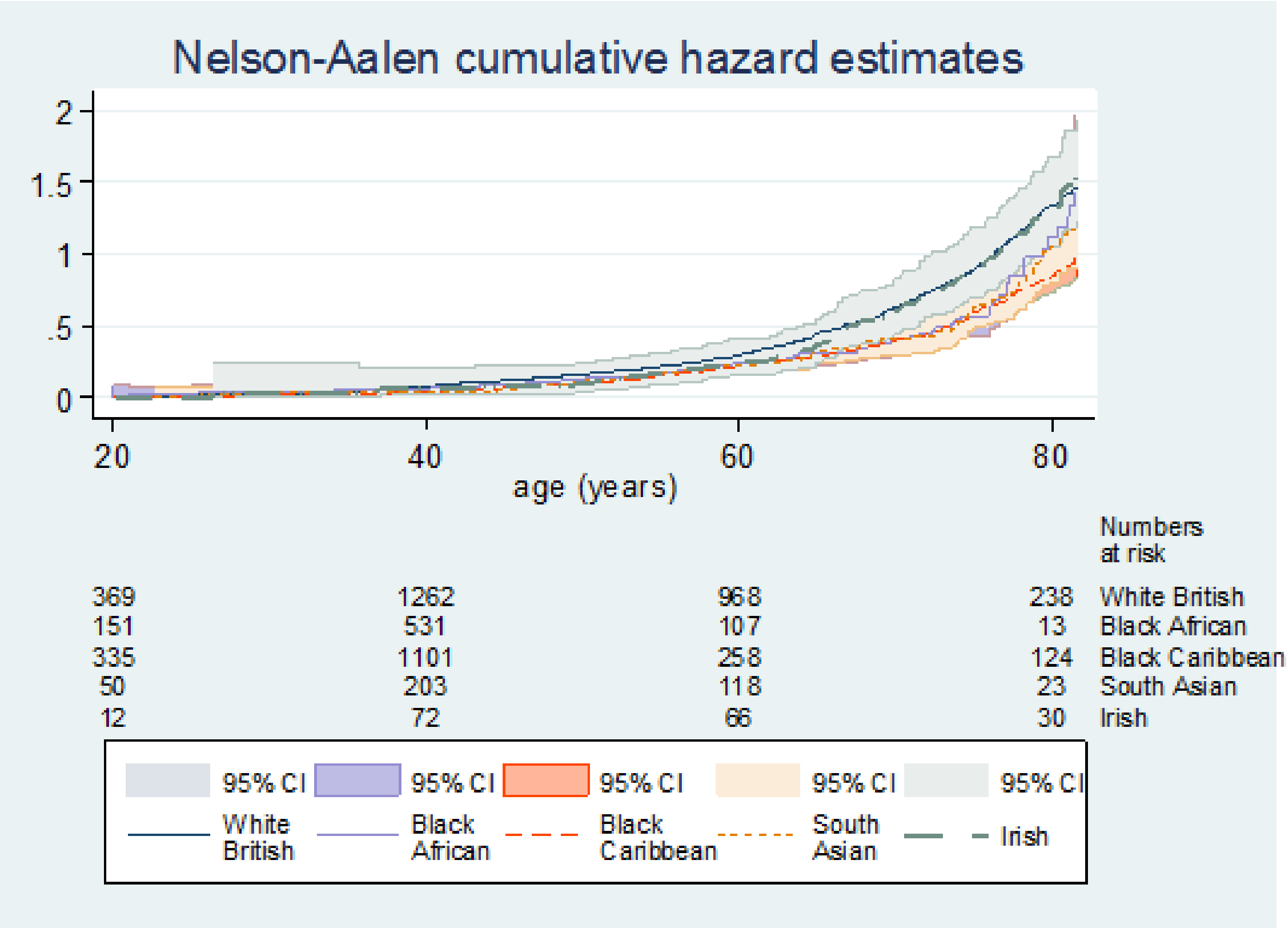

Supplementary material

Table 1: Sub-Hazard Ratios (sHRs) for all-cause mortality- sensitivity analysis[1]

				**Time since diagnosis**						
				**0-3 years**		**3-7 years**		**7 years+**		
		**Total cases**	**Deaths**	**Adjusted sHR (95% CI)**	**P value**	**Adjusted sHR (95% CI)**	**P value**	**Adjusted sHR (95% CI)**	**P value**	**P value**
Ethnicity	White British	9278	1142	REF		REF		REF		
	Black Caribbean	4946	334	0.49 (0.40, 0.60)	<0.0001	0.94 (0.70, 1.27)	0.69	0.60 (0.53, 0.67)	<0.0001	<0.0001
	Black African	2573	107	0.60 (0.53, 0.67)	<0.0001	0.77 (0.62, 0.95)	0.014	0.49 (0.40, 0.60)	<0.0001	
	South Asian	1279	96	0.67 (0.55, 0.82)	<0.0001	0.50 (0.37, 0.66)	<0.0001	0.67 (0.55, 0.82)	<0.0001	
	Irish	565	107	1.05 (0.88, 1.27)	0.57	1.17 (0.81, 1.71)	0.40	1.05 (0.88, 1.27)	0.57	
		**Total cases**	**Deaths**	**Adjusted sHR (95% CI)**						
Gender	Male	9856	920	REF						
	Female	8785	866	0.85 (0.78 ,0.93)	<0.0001					<0.0001
Diagnosis	Non-affective psychosis	13476	1370	REF						
	Affective psychosis	5165	416	0.76 (0.68, 0.84)	<0.0001					<0.0001
Marital status	Married/ cohabiting	2842	272	REF						
	Divorced/single/separated	15799	1514	1.24 (1.09, 1.41)	0.001					0.0013
Co-morbid substance misuse	None	15409	1535	REF						
	Present	3232	251	1.05 (0.92, 1.20)	0.48					0.48
Area-level Deprivation	Least deprived	3749	379	REF						
	2	3712	344	0.89 (0.77, 1.02)	0.10					0.19
	3	3725	316	0.85 (0.74, 0.98)	0.030					
	4	3771	376	0.91 (0.79, 1.05)	0.19					
	Most deprived	3684	371	0.87 (0.76, 1.00)	0.044					

[1]Sensitivity analysis: sub-Hazard ratios (sHRs) for association of each variable with all-cause mortality with emigrations out of the cohort specified as a competing risk, Wald tests used to assess strength of associations, estimates are adjusted for age plus all other variables shown in table

Supplementary material

Table 3: Adjusted Hazard Ratios for all cause mortality[1]

		Total cases	Deaths	Adjusted HR (95% CI) Diagnosis, affective psychosis vs. non-affective	P value (Wald)	Time since diagnosis 0-3 years	P value (Wald)	3-7 years	P value (Wald)	7 years+	P value (Wald)	P value (LRT)
Ethnicity	White British	9278	1142			REF		REF		REF		
	Black Caribbean	4946	334			0.43 (0.29, 0.65)	<0.0001	0.72 (0.51, 1.03)	0.071	0.83 (0.59, 1.16)	0.28	0.0060
	Black African	2573	107			0.69 (0.36, 1.31)	0.25	0.72 (0.38, 1.37)	0.32	0.46 (0.24, 0.89)	<0.021	
	South Asian	1279	96			0.63 (0.35, 1.12)	0.12	0.63 (0.36, 1.10)	0.10	0.66 (0.37, 1.18)	0.16	
	Irish	565	107			1.19 (0.81, 1.75)	0.37	1.33 (0.93, 1.92)	0.12	1.14 (0.78, 1.68)	0.49	
Ethnicity	White British	9278	1142	0.84 (0.73, 0.96)	0.0090							0.026
	Black Caribbean	4946	334	1.07 (0.78, 1.46)	0.67							
	Black African	2573	107	0.90 (0.49, 1.63)	0.72							
	South Asian	1279	96	0.75 (0.44, 1.26)	0.27							
	Irish	565	107	0.62 (0.39, 0.99)	0.047							
Gender	Male	9856	920	REF	REF							0.0029
	Female	8785	866	0.86 (0.78, 0.95)	0.0030							
Marital status	Married/ cohabiting	2842	272	REF	REF							0.0089
	Divorced/single/separated	15799	1514	1.19 (1.04, 1.36)	0.010							
Co-morbid substance misuse	None	15409	1535	REF	REF							0.016
	Present	3232	251	1.19 (1.03, 1.37)	0.015							
Area-level Deprivation	Least deprived	3749	379	REF	REF							0.28
	2	3712	344	0.90 (0.77, 1.04)	0.16							
	3	3725	316	0.88 (0.76, 1.03)	0.10							
	4	3771	376	0.90 (0.78, 1.04)	0.17							
	Most deprived	3684	371	0.99 (0.86, 1.15)	0.91							

*[1]Multivariable Cox regression model with ethnicity*time interaction and ethnicity*diagnosis interactions. Estimates are adjusted for all other variables displayed in the table. P value from Likelihood Ratio Tests (LRT) assessing the overall interaction of ethnicity*diagnosis was p=0.39*
